# Magnetic Resonance Spectroscopy in ALS

**DOI:** 10.3389/fneur.2019.00482

**Published:** 2019-05-10

**Authors:** Sanjay Kalra

**Affiliations:** Division of Neurology, Department of Medicine, Neuroscience and Mental Health Institute, University of Alberta, Edmonton, AB, Canada

**Keywords:** biomarker, magnetic resonance spectroscopy, neuroimaging, amyotrophic lateral sclerosis, neurodegeneration

## Abstract

Proton magnetic resonance spectroscopy (MRS) provides a means of measuring cerebral metabolites relevant to neurodegeneration *in vivo*. In amyotrophic lateral sclerosis (ALS), neurochemical changes reflecting neuronal loss or dysfunction (decreased N-actylaspartate [NAA]) is most significant in the motor cortex and corticospinal tracts. Other neurochemical changes observed include increased myo-inositol (mIns), a putative marker of gliosis. MRS confirmation of involvement of non-motor regions such as the frontal lobes, thalamus, basal ganglia, and cingulum are consistent with the multi-system facet of motor neuron disease with ALS being part of a MND-FTD spectrum. MRS-derived markers exhibit an encouraging discriminatory ability to identify patients from healthy controls, however more data is needed to determine its ability to assist with the diagnosis in early stages when upper motor neuron signs are limited, and in distinguishing from disease mimics. Longitudinal change of NAA and mIns do not appear to be reliable in monitoring disease progression. Technological advances in hardware and high field scanning are increasing the number of accessible metabolites available for interrogation.

## Background and Technical Considerations

Magnetic resonance imaging has emerged as a promising tool to provide a biomarker in neurological and psychiatric disorders. Routine structural MRI is not helpful in this regard in ALS as signal intensity and gross volume changes in T1 and T2 weighted images is not apparent in the vast majority of cases (1). Advanced imaging and post-processing methods are necessary to reveal pathology that is not evident to the naked eye. Numerous studies have demonstrated the potential of MRS in research and clinical care in brain disorders, including ALS. Results have been consistent amongst investigators using different methods to quantify key metabolites such as NAA, and renewed interest along with advancing technology are leading to studies probing previously inaccessible chemicals such as Gama-aminobutyric acid (GABA).

With routine structural MRI, the abundance and microenvironment of protons is quantified resulting in essentially images of the distribution of water since it is the most abundant proton-rich molecule. The most basic MRS experiment quantifies instead protons in molecules other than water. The experiment is usually a measurement from a defined volume (rather than the whole brain), and produces a spectrum rather than an image. Different peaks in the spectrum arise from different protons and their microenvironment. The positioning along the x-axis of peaks is dependent on the spin frequency of the protons contributing to the peak, with the area under the peak dependent on the number of protons. Small shifts in frequency can occur due to magnetic field perturbations arising from nearby molecules, leading to a change the shape of a peak (singlets, doublets, triplets, etc.). The frequency of a peak and its splitting structure are key elements used in the identification of the metabolite from where the peak arises.

Images can be produced from metabolites such as NAA, however these are of much lower resolution than structural MRI (which is essentially MRS of water) because of the very low concentrations of such molecules. The lower concentration of the target metabolites also means that MRS scans are comparatively longer than routine structural imaging. Rather than a structural evaluation, MRS is a means of quantifying neurochemistry in the brain of low abundance metabolites. MR spectra can be obtained using other nuclei, including phosphorus, fluorine, carbon, and sodium. These typically require alternate hardware (e.g., specific RF coils) to that typically available with clinical and clinical research systems used for proton MRS.

### Metabolites

The metabolites that are visible and quantifiable is dependent on a number of factors, and requires a sufficient concentration typically in the range of micromoles/gm. Spectral resolution and SNR must be sufficient to accurately identify and quantify individual peaks, and this is determined by many factors including B0 field strength and homogeneity, acquisition sequence (PRESS, STEAM, MEGA-PRESS, etc.), and TE, amongst others. Higher field strengths and lower TE in general give access to more metabolites.

**Figure 1:**
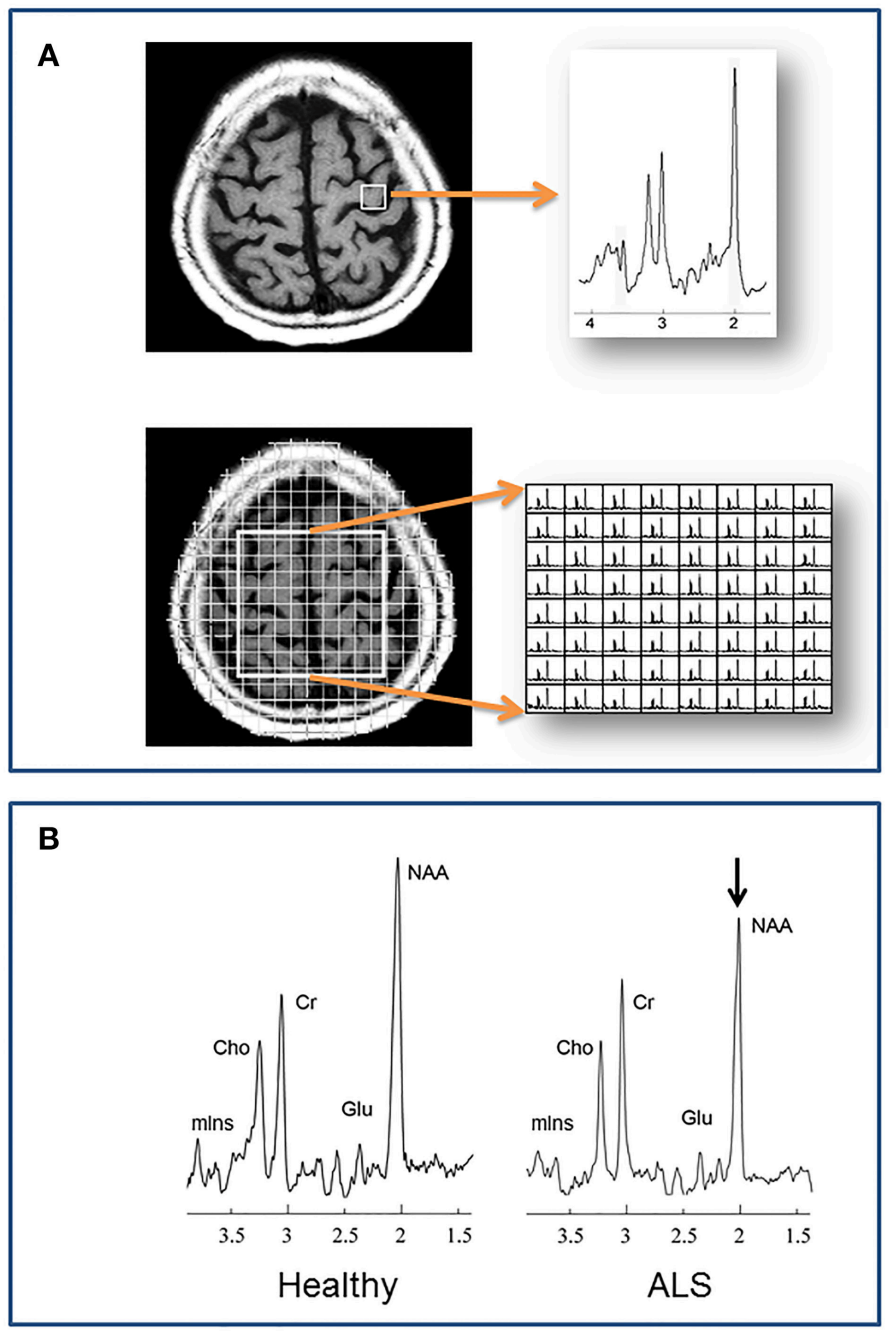
**(A)** Localization methods. Neurochemical data are acquired from specified volumes during a single MRS scan. A single spectrum is recorded in single voxel spectroscopy (SVS), such as from the left precentral gyrus in the example at top. With magnetic resonance spectroscopic imaging (MRSI) multiple spectra are acquired, such as from a 2 dimensional plane centered over the central sulcus in the example at bottom. **(B)** A representative spectrum from the motor cortex of a healthy individual compared to one from a patient with ALS. N-acetylaspartate is reduced in ALS, reflecting reduced neuronal integrity. Cho, choline; Cr, creatine; Glu, glutamate; mIns, myo-inositol; NAA, N-acetylaspartate.

There are a number of metabolites detectable using contemporary methods that have relevance in neurological disease (Figure 1). N-acetylaspartate (NAA), along with a small contribution from N-acetylaspartylglutamate, is localized only in neurons and their processes, and thus NAA serves as a marker of neuronal integrity. The total creatine peak arises from metabolites (creatine plus phosphocreatine) involved in energy metabolism. Total choline (choline, phosphorylcholine, glycerophosphorylcholine) is a marker of membrane turnover. Increased levels are reported with cell proliferation, both neuronal and glial.

Beyond NAA, there are a number of metabolites that can be measured which are of particular relevance to neurodegeneration in ALS. Myo-inositol (mIns) has a preferential distribution in glial cells, and is as such a putative glial marker. Glutamate is the primary CNS excitatory neurotransmitter. It is difficult to separate using routine MRS techniques from glutamine, and is thus may be expressed as “Glx.” GABA is the primary inhibitory neurotransmitter in the brain. Glutathione functions as an antioxidant. Glutamate, GABA, and glutathione can be measured at ultrahigh field (7 T), or high field (3 T) using advanced spectral editing methods.

### Acquisition

MRS can be performed using the same hardware systems as for structural imaging. The lowest field strength advised, and indeed what many papers to date report experiments from, are studies at 1.5 T. The benefits of high field imaging include access to more metabolites, shorter acquisition times, and higher spatial resolution. The former comes from increased SNR and increased chemical shift dispersion. The benefit is particularly relevant to detecting metabolites that have very low concentration or a complex resonance peak structure such as glutamate and GABA.

Unlike whole-brain structural imaging the location from where a spectrum is acquired usually must be pre-defined. Traditional localization schemes to define where spectra are acquired, include single voxel spectroscopy (SVS) and multivoxel spectroscopic imaging (MRSI) (Figure 1). In the former, a single spectrum is acquired from a discrete volume of interest (VOI), such as the motor cortex, internal capsule, etc. With MRSI, individual spectra are acquired from multiple regions within a 2-dimensional slab or a 3-dimensional volume. These volumes are positioned at the time of scanning, and the acquired spectra within the volume are selected after processing. Critical steps during data acquisition, but beyond the scope of this review, are water and lipid suppression, and shimming to minimize local field inhomogeneity.

### Data Post-processing and Quantification

Post-processing of data includes a number of steps (e.g., residual water suppression, Fourier transformation, phase correction), with ultimately the production of spectral peaks. These are baseline corrected and fitted. The area under a fitted peak correlates with the number of protons contributing to the signal and thus metabolite density. Processing and quantification is available on MRI consoles, or with stand-alone software such as LCModel (2).

It is paramount to be aware that a metabolite resonance reflects its contributing protons throughout the voxel being sampled, including all cell and tissue types (neurons, glia, gray matter, white matter) and compartments (intracellular, extracellular). The derivation of absolute concentrations (i.e., mmol/L) requires additional MR experiments and processes to correct, for example, partial volume effects, coil loading, field inhomogeneity, and relaxation effects with a potential concern to data reliability. Resonance signals are thus often reported as a ratio to a reference metabolite, such as Cr or Cho (NAA/Cr); this inherently performs the aforementioned correction, however it requires the assumption that the reference metabolite is stable in the disease under question. Normalization with a water signal is used by some as an alternative and obviates the issue of whether Cr or Cho are unchanged, though comes with its own issues.

### Recent Advances

#### High and Ultrahigh Field Imaging, and “New Metabolites”

Within the last decade, research and clinical MR systems have transitioned from a low field of 1.5 T to a high field of 3 T. Studies at the latter are becoming common place, with studies at the ultrahigh field of 7 T emerging.

The benefits of high field imaging include access to more metabolites, shorter acquisition times, and higher spatial resolution. The former comes from increased SNR and increased chemical shift dispersion; this is particularly relevant to metabolites that have very low concentration or complex resonance peak structure such as glutamate, GABA, and mIns. Higher field systems are accompanied by a number of challenges that require attention for successful spectroscopy experiments: greater main (B0) and applied RF (B1) field inhomogeneity and chemical shift mis-registration, altered T1 and T2 relaxation times, greater safety concerns, and higher purchase and operating costs (3).

#### 3D MRSI and Automated Quantification

Single voxel spectroscopy and MRSI constrain the acquisition of data from small and discrete regions (volume of interest). These spatial restrictions are necessary, in part, for optimization of field homogeneity. Thus, MRS scans demand an additional level of knowledge, expertise, and experience from the MR technologist required for accurate positioning of the VOI. Larger sampling of the brain can be done with multislice MRSI (4–6), or 3D MRSI (7), however these further increase acquisition times. Echo-planar spectroscopic imaging (EPSI) has been an exciting development as it permits high resolution volumetric (whole brain) spectroscopic imaging in a single acquisition within a clinically acceptable timeframe (8). It has been applied in ALS to study the neurochemistry of the CST in its 3-dimensional extent (9, 10), and of multiple spatially discrete areas (11, 12).

## Results

At the time of writing, a general survey reveals there have been just over 60 papers published describing human proton MRS experiments in ALS, with inclusion of ~1,400 patients with ALS or related motor neuron disease (primary lateral sclerosis, progressive muscular atrophy). The majority of papers have interrogated neurochemistry of the motor system, namely the primary motor cortex and corticospinal tract. Published works also report findings in “extra-motor” regions including the prefrontal cortex, subcortical gray, brainstem, and spinal cord. Longitudinal MRS studies are few, as they are with other imaging modalities. With few exceptions, studies published since 2011 have been done at high field (3 T) or ultrahigh field (7 T).

Participants in studies have consisted of patients meeting El Escorial Criteria for ALS with combined upper and lower motor neuron signs. The number of MND participants in each study range from 7 to 169, with many studies having 10–30. Some have included subjects with no UMN signs (PMA) (6, 11, 13–16) generally showing the expected correlation of more normal NAA in such subjects. All studies have been conducted at a single center, except for a prospective multicenter study conducted at 4 sites in the Canadian ALS Neuroimaging Consortium [ClinicalTrials.gov # NCT02405182 and in press (Neurology: Clinical Practice)].

### Cross-Sectional

#### Motor Cortex

The regional focus of most studies has been on the motor cortex or CST. NAA ratios to Cr, Cho, or Cr+Cho are reduced in the precentral gyrus (4–6, 12, 13, 15, 17–39). A decline in absolute quantities of NAA (14, 16, 21, 22, 31, 40–43) corroborate these observations of reduced ratios of NAA. A gradient effect can be observed when spectra are acquired from the motor cortex and regions immediately surrounding it, such that less prominent reductions are present in the postcentral gyrus and premotor areas compared to the precentral gyrus (13, 25).

#### Corticospinal Tract

The corticospinal tract has been interrogated using various methods. One group found reduced NAA/Cr+Cho) in the centrum semioval (CSO) and internal capsule combined, but not individually in these two regions (4). In part contrary to this, a study using a coronal MRSI method in the plane of the CST found reduced NAA/Cr in the precentral gyrus and corona radiata, but normal levels in the internal capsule and cerebral peduncle (44). Another found reduced NAA/Cr in both the motor cortex and IC (32). NAA of the entire CST was found to be reduced using a whole-brain 3D spectroscopic acquisition protocol (9, 10).

#### Extra-Motor Regions

The presence of frontotemporal lobar degeneration (FTLD) is supported by reduced NAA indices in various frontal regions including the dorsolateral (11, 23) and mesial prefrontal (19, 45) cortices. Mesial prefrontal cortex neurochemistry is abnormal in patients who for the most part are not cognitively impaired, suggesting MRS may be more sensitive to detecting FTLD than clinical measures (45). “Extra-motor” degeneration was similarly demonstrated in the mid-cingulate gyrus (34), thalamus (34, 46), and basal ganglia (46). As expected, NAA is normal in the parietal and occipital lobes (5, 11, 23, 25, 26, 33, 40) and cerebellum (14).

#### Brainstem

Reductions in NAA indices are described by most (21, 43, 47, 48) but not all studies (33) that have examined the brainstem.

#### Spinal Cord

MRS of the upper cervical spinal cord revealed substantially reduced NAA ratios 25–40% in patients with ALS (49, 50). Notably, a single voxel was used enclosing the breadth of the cord. Thus, the spectrum included contributions from both white matter tracts and the anterior horn and other cells in the gray matter. One group extended their methods to investigate neurochemical changes in asymptomatic SOD1+ individuals (51). They found comparably reduced NAA/Cr and NAA/mIns in asymptomatic (39.7% and 18.0%) and patients with ALS (41.2% and 24.0%) compared to healthy controls, inferring the presence of neurochemical changes early in the disease and even before symptoms or signs are present.

#### Other Metabolites

Reflective of astrogliosis, mIns is increased in the motor cortex (29, 40, 43, 48, 52, 53). The NAA/mIns ratio may be a more robust marker of degeneration as it reflects the combined pathology of decreased neuronal integrity and gliosis with the individual metabolite levels becoming abnormal in opposite directions in the motor cortex (16, 29, 48) and mesial prefrontal cortex (45).

Given one of the putative pathophysiological mechanisms is excitotoxicity, one may have expected Glu to be increased. However, results have been conflicting for the motor cortex where it (or Glx) were normal (16, 21, 43, 52), increased (32), or decreased (40). Studies at 7 T where its quantification may be more precise were conflicting with levels in the motor cortex normal (48) or increased (53). Glx was increased in the medulla (54) along with a negative correlation with the ALSFRS bulbar subscore. Later studies of the pons revealed normal pontine Glu or Glx (43, 48). MRS measurements of the inhibitory neurotransmitter GABA in the motor cortex have been reported to be reduced using the MEGA-PRESS technique at 3T (43, 55), but normal using a STEAM sequence at 7T (53). As discussed above MRS measurements will largely reflect the intracellular metabolic rather than synaptic neurotransmitter pool; as such, reductions may simply be the result of neuronal loss.

Initial findings of decreased glutathione in the primary motor cortex (35) which would have been supportive of a role for oxidative stress in the pathogenesis of ALS were not replicated by subsequent studies at 3 T or 7 T (48, 53).

### Diagnostic Accuracy

A number of studies have assessed the discriminatory power of NAA and its ratios in the motor cortex to separate ALS patients from healthy controls. Sensitivity ranges from 53 to 100%, specificity ranges from 37 to 100%, with the average amongst the studies ~80% for both. MRS improves the accuracy when combined DTI assessment of the corticospinal tract (56, 57) or of signal change on structural imaging (36, 56).

### Longitudinal

A number of studies suggest a decline in NAA indices over varying intervals; interpretation of these reports is difficult due to small numbers of patients (5, 19, 22, 52, 58, 59).

In a more rigorous design, longitudinal change in absolute NAA and its ratio to Cr and Cho were measured every 3 months out to 1 year. Changes were seen in the motor cortex and outside the motor cortex over 3 and 9 months, respectively depending on the El Escorial designation, but overall did not follow a consistent pattern (27). In a treatment trial of growth hormone, the placebo arm of 20 patients did not have any change in motor cortex NAA/(Cho+Cr) at 0, 6, or 12 months (60).

In a larger study of 43 patients, 30 had at least one follow up scan on a 3 month interval, demonstrating a non-significant (*p* = 0.06) decline in motor cortex NAA/Cr (6).

Recently, longitudinal neurochemical observations were made at 7 T at 6 and 12 months. Motor cortex NAA/mIns declined and pontine Glx increased. In a sub group analysis, this pattern of neurochemical change was not present in those whose upper limb and bulbar function did not deteriorate over time (61).

### Correlations

The presence of correlations with an imaging finding provides a degree of biological validity to the imaging metric. Not surprisingly, NAA indices are more reduced in patients with a greater severity of UMN findings on neurological examination (13–15, 20, 28, 31, 40, 52), however this is not always the case (48). As a measure of UMN function, finger tapping has the advantage of being objective and providing a continuous measure. Correlations with tapping have been reported in a number (4, 6, 16, 18, 39), but not all, (44) studies. A few studies have also noted a correlation with the El Escorial criteria (15, 27, 48). Reports are conflicting with respect to associations with disease duration, progression rate, or disability as quantified by ALSFRS-R. With respect to the latter this is not surprising given that disability is largely driven by muscular weakness which in turn is dependent considerably on LMN status.

The evaluation of neurochemical associations with cognitive or behavioral impairment is limited in ALS. As would be expected dorsolateral prefrontal cortex NAA/Cr correlates with cognitive measures of executive function, including verbal fluency (11) and the Wisconsin Card Sorting Test (23). However, mesial prefrontal cortex NAA/mIns did not correlate with the Addenbrook Cognitive Examination or verbal fluency (45); this may have been due to the localization of the voxel (mesial rather than dorsolateral) or that the ACE may not be an optimal cognitive screening measure in ALS (62).

The marked clinical heterogeneity of patients with ALS makes prognostication a difficult task, yet this would be extremely helpful in clinic for counseling patients and to assist as an enrichment strategy in clinical trials. MRS was the first neuroimaging modality to reveal an association of cerebral degeneration with survival. Reduced motor cortex NAA/Cho was the strongest predictor of shorter survival, followed by older age and shorter symptom duration (30).

## Monitoring Treatment

There have been several studies evaluating treatment effects using MRS. The commencement of riluzole, an antiglutamatergic agent, is accompanied by an increase in NAA/Cr in the motor cortex observed at 1 day (63) and 3 weeks (58) after its initiation. Increases in NAA/Cr suggest the existence of a population of metabolically dysfunctional neurons amenable to treatment. This supposition is supported by the observation of maintained NAA/Cr levels in ALS patients in contrast to a decline in NAA/Cr in healthy controls who received creatine supplementation (24). Changes in NAA/Cr were not observed with gabapentin (25), intrathecal BDNF (26), or minocycline (64). Preliminary observations have also been made on the Glx signal with creatine supplementation (24, 65). In contrast to the studies discussed thus far, there have been reports from studies that have performed sub-analyses on patients comparing those who are taking riluzole to those who are riluzole-naïve (43, 48); these have had varying results.

## Conclusions and Future Directions

What has MRS delivered in the field of ALS thus far, and what is needed?

Cross sectional changes reflecting cerebral neuronal impairment (abnormal NAA indices) are consistently present, and with reasonable accuracy in discriminating patients from controls in group analysis. However, with regards to diagnostic utility, a biomarker of cerebral degeneration will be most helpful in the clinic for patients presenting with LMN signs but insufficient UMN signs; MRS data (as for much of the neuroimaging field) is lacking for such patients. In the more immediate future, MRS should be able to play a part in addressing phenotypic heterogeneity, as associations have been demonstrated with various behavioral measures. Future studies addressing diagnostic potential and heterogeneity would benefit from larger sample sizes, deep phenotyping, inclusion of disease mimics, incorporation of other imaging modalities (e.g., DTI), and incorporation of biofluids for correlative and validation analyses. Of note, there is very little known of the association of cerebral neurochemicals with cognitive impairment in ALS.

There is sensitivity to measuring longitudinal change in metabolites that appears best observed with time intervals of at least 3 months. However, there is considerable variability, which currently prohibits its use as a biomarker of disease progression. The experience of MRS to date of assessing response to therapy has been largely proof of principle. Progress in this area has been hampered in part by the lack of robust disease modifying therapies upon which to frame spectroscopy experiments. Inclusion of MRS in phase II clinical trials may provide opportunities validating metabolites as measures of disease progression, target engagement, or therapeutic response.

The feasibility for MRS to be applied for clinical and routinely for research applications, especially for multicenter efforts and to allow inter-study comparison of results, will require refinement, optimization, and standardization of acquisition and processing protocols, in parallel with greater user expertise. Reference to general (66) and ALS-specific guidelines (67) are starting points for such an endeavor. The advent of whole brain MRSI combined with automated quantification is a significant advancement that could facilitate the modality's uptake to more research labs and eventually clinics.

Advances in technology (higher fields, new sequences) are already permitting the quantification of previously undetectable disease-relevant metabolites and of anatomical regions previously inaccessible (spinal cord). This will continue to provide opportunities for exploring biological insights *in vivo* and for evaluating novel disease markers that may meet the desperate need of a biomarker in ALS.

## Author Contributions

The author confirms being the sole contributor of this work and has approved it for publication.

### Conflict of Interest Statement

The author declares that the research was conducted in the absence of any commercial or financial relationships that could be construed as a potential conflict of interest.
